# Sensory Variability in Propolis from the Mexican Comarca Lagunera: A Multivariate Analysis

**DOI:** 10.3390/foods15142450

**Published:** 2026-07-10

**Authors:** Perla Susana Martínez-Rojas, Rafael García-Vázquez, Blanca Isabel Sánchez-Toledano, Marco Andrés López-Santiago, Ramón Trucíos-Caciano, Julián Cerano-Paredes, Miguel Ángel Mata-Espinosa, Lorenzo Danilo Granados-Rivera

**Affiliations:** 1 Arid Zones Regional University Unit (URUZA), Universidad Autónoma Chapingo (UACh), Bermejillo 35230, Durango, Mexicogarciarafael154@gmail.com (R.G.-V.);; 2 Campo Experimental Zacatecas, National Institute of Forestry, Agricultural and Livestock Research (INIFAP), Calera 98500, Zacatecas, Mexico; 3 National Disciplinary Research Center-Water-Soil-Plant-Atmosphere Relationship (CENID-RASPA), Instituto Nacional de Investigaciones Forestales, Agricolas y Pecuarias (INIFAP), Gómez Palacio 35140, Durango, Mexico; 4 Campo Experimental Terán, Instituto Nacional de Investigaciones Forestales, Agricolas y Pecuarias (INIFAP), General Terán 67400, Nuevo León, Mexico

**Keywords:** propolis, sensory evaluation, Comarca Lagunera, organoleptic attributes

## Abstract

Propolis has gained widespread interest due to its multiple applications in the food and pharmaceutical industries. Therefore, the objective of this study was to determine the sensory differences among propolis samples collected in the Mexican Comarca Lagunera region and to identify the sensory attributes highest-rated by a trained sensory panel. The methodology involved collecting ten propolis samples following the guidelines of the Mexican Official Standard NOM-003-SAG/GAN-2017. A panel of 24 panelists was established, evaluating the samples using an attribute-specific categorical scale and a nine-point hedonic Likert scale. The attributes considered were color, aroma, taste, consistency, and overall acceptance. Data were analyzed using descriptive statistics, Spearman correlation coefficients, hierarchical cluster analysis, Principal Component Analysis (PCA), and the non-parametric Kruskal–Wallis test. Results indicated that taste and aroma were the most relevant sensory attributes, predominantly characterized by a bitter taste and intense aroma. Both attributes showed statistically significant differences. Samples TLA1 and SB1 obtained the highest scores in these parameters. Conversely, SB2 and SB3 showed lower acceptability across all evaluated attributes. This study highlights the need for continued multidisciplinary research aimed at the sensory characterization of propolis and at strengthening its regional documentation. Such efforts will contribute to its commercial valuation, geographical differentiation, and consolidation as a bee product with high functional potential.

## 1. Introduction

Propolis is a natural resinous substance produced by bees, globally recognized for its anti-inflammatory, antitumor, antibacterial, antifungal, antiviral, and antioxidant properties [1,2]. Therapeutic applications have been demonstrated across various clinical fields, targeting conditions such as metabolic, dermatological, respiratory, gastrointestinal, cardiovascular, and gynecological diseases, as well as COVID-19 [2].

Globally, propolis and its derivatives represent a growing market [3]. This growth has sparked increasing interest in its use in food products and as an active ingredient in biocosmetics, driving efforts to improve both quality and production volume [1,4].

In Mexico, research has focused mainly on the biological properties of propolis and the influence of geographic and climatic conditions on its chemical composition [1,5,6,7,8]. However, exploring its commercial potential as a value-added product is required.

The Comarca Lagunera region, located in northern Mexico, is distinguished by its apicultural sector. This industry, which contributes to agricultural crop pollination and the production of honey derived from mesquite flowers, has established a strong presence in both national and international markets [9]. It is estimated that nearly 50% of this honey production is exported to the United States and European countries, while the remainder is distributed in regional and national markets, underscoring the socioeconomic importance of this sector for the region [10].

Despite the socioeconomic relevance of beekeeping in the Comarca Lagunera, propolis remains under-researched in this region. The area’s characteristic arid climate, combined with its unique desert microphyllous scrub vegetation—composed mainly of honey mesquite and wild flora [11]—is likely to confer unique sensory attributes to the local propolis. Investigating these sensory differences represents a critical first step toward addressing the current lack of information on propolis from northern Mexico. Furthermore, this characterization lays the groundwork for future geographical differentiation, providing added value and an economic alternative for local beekeepers, who currently rely primarily on honey production [10].

Scientific approaches to propolis analysis are highly diverse, leading to a fragmented research landscape; consequently, despite being a highly beneficial product, its full potential has not yet been harnessed [12].

In this context, research dedicated exclusively to the sensory evaluation of raw propolis remains in its early stages. Although previous literature has addressed sensory analysis, studies have predominantly focused on the incorporation of propolis into other products [13,14,15]. This trend responds to the growing interest in integrating it as an active ingredient in the food, cosmetic, and pharmaceutical industries [16].

Nevertheless, to guarantee sensory quality during manufacturing and in the development of propolis-based products, it is essential to directly characterize its sensory profile and establish quality standards through the identification of differentiating attributes. Therefore, the objective of the present research was to determine the sensory differences among propolis samples collected from the Comarca Lagunera region and to identify the sensory attributes highest-rated by a trained sensory panel.

## 2. Materials and Methods

### 2.1. Study Area

The study was conducted in the Comarca Lagunera, a region located in the center of Durango and Coahuila states in northern Mexico [17]. According to the National Institute of Forestry, Agricultural, and Livestock Research (INIFAP, by its Spanish acronym) [18], this zone is comprises 15 municipalities: in the state of Durango, it includes Lerdo, Gómez Palacio, Mapimí, Nazas, Rodeo, Tlahualilo, General Simón Bolívar, San Juan de Guadalupe, San Luis del Cordero, and San Pedro del Gallo; in the state of Coahuila, it covers Matamoros, San Pedro, Torreón, Viesca, and Francisco I. Madero.

Beekeeping in the Comarca Lagunera is practiced in agricultural areas adjacent to wild vegetation, promoting diversified apiculture. The main production centers are located in Gómez Palacio and Matamoros, where an ecological mosaic of desert microphyllous scrub and secondary vegetation predominates [19]. Mesquite (*Prosopis* spp.) is the most economically relevant wild species, while crops such as alfalfa, annual forages, and horticultural crops constitute the primary food sources for honeybees (*Apis mellifera*) [19].

### 2.2. Propolis Samples Collection

Propolis collection was carried out in accordance with the guidelines established in the Mexican Official Standard NOM-003-SAG/GAN-2017 regarding production and processing specifications (Official Gazette of the Federation [DOF, by its Spanish acronym]) [20]. To guarantee quality and avoid contamination of the resinous material, plastic collecting grids were used as specified by the regulation, minimizing contact with potential toxic or environmental contaminants. A total of ten samples were obtained from different locations in the Comarca Lagunera through the voluntary cooperation of local beekeepers. The samples were coded according to their geographical origin and the corresponding harvest number:

1. BER1 (Bermejillo, Mapimí, 1st harvest)/25°53′17″ N, 103°37′20″ W
2. ECM1 (El Coyote, Matamoros, 1st harvest)/25°41′40″ N, 103°17′08″ W
3. ECM3 (El Coyote, Matamoros, 3rd harvest)/25°41′40″ N, 103°17′08″ W
4. MAT1 (Matamoros, 1st harvest)/25°31′41″ N, 103°13′42″ W
5. MAT3 (Matamoros, 3rd harvest)/25°31′41″ N, 103°13′42″ W
6. SB1 (Simón Bolívar, 1st harvest)/24°39′39″ N, 103°13′41″ W
7. SB2 (Simón Bolívar, 2nd harvest)/24°39′39″ N, 103°13′41″ W
8. SB3 (Simón Bolívar, 3rd harvest)/24°39′39″ N, 103°13′41″ W
9. TLA1 (Tlahualilo, 1st harvest)/26°06′34″ N, 103°26′27″ W
10. TOR3 (Torreón, 3rd harvest)/25°32′38″ N, 103°27′00″ W

Specific details regarding collection dates and environmental conditions for each sample are presented in Supplementary Table S1. Due to the lack of specific biological data on the seasonality of propolis production in northern Mexico, an operational grouping strategy was adopted based on the established local beekeeping calendar. In the Comarca Lagunera, the spring season is characterized by the intense and predominant blooming of mesquite (*Prosopis* spp.), which defines the first harvest [10]. During the summer and autumn-winter periods, resource availability becomes multifloral, sustained by the blooming of wild secondary vegetation (such as aceitilla), late-blooming species like tamarisk (*Tamarix aphylla*), and agricultural crops [10]. Based on these flowering dynamics, the propolis samples were grouped into three periods: harvest 1 (Mesquite/spring), harvest 2 (Multifloral/summer), and harvest 3 (Multifloral/autumn-winter). The number of hives monitored per apiary ranged from three to five, depending on the site’s production capacity, yielding between 30 and 45 g of propolis per grid.

To ensure the representativeness of the floral environment, apiaries were separated by at least 3 km, in accordance with the average foraging range of honeybees (*A. mellifera*). Once collected, the samples were placed in dark bags, labeled, and any visible impurities that could interfere with the sensory analysis, such as bee remains, plant fibers, and other foreign matter, were manually removed.

### 2.3. Sample Handling and Preparation

Following the recommendations established in NOM-003-SAG/GAN-2017, sample handling was performed under controlled sanitary conditions, using nitrile gloves and face masks, to preserve physicochemical integrity and avoid cross-contamination during the cleaning and fractionation process [20]. Visible impurities were manually removed without subjecting the propolis to thermal or chemical treatments that could alter its properties. The samples were individualized for sensory evaluation and stored under refrigerated conditions (−20 °C); the integrity of the material was maintained until the moment of its analysis by the sensory panel.

Subsequently, ethanolic extracts (10% *w*/*v*) were prepared by macerating 10 g of frozen propolis in 100 mL of 70% ethanol for seven days at room temperature (20–22 °C) in the dark, followed by filtration using Whatman No. 1 filter paper. All samples were prepared following the same protocol. Propolis samples were extracted with ethanol because it effectively solubilizes the volatile and phenolic compounds responsible for the perception of aroma and taste, providing a homogeneous matrix for sensory comparison. Although ethanol may contribute to the aromatic background, the purpose of its use was to compare extracts prepared under identical conditions rather than to quantify the perception of ethanol itself.

### 2.4. Formation of the Sensory Panel and Attributes Evaluated

The sensory analysis of the propolis samples was conducted in the Sensory Analysis Laboratory located at INIFAP, Zacatecas Experimental Field, Mexico (22°54′ N, 102°39′ W; altitude 2197 masl). The facility has 12 tasting booths aligned with International Organization for Standardization (ISO) standards, which guaranteed food safety and contaminant control through the ISO 22000:2018 standard [21].

Based on the guidelines of the Secretariat of Agriculture and Rural Development (SADER) in the National Atlas of Bees and Bee Products [22], as well as in NOM-003-SAG/GAN-2017, the evaluated attributes included color, aroma, taste, and consistency. To deepen sensory perception, an additional attribute termed “overall acceptance” was incorporated. These attributes are considered fundamental for both industrial processing and consumer organoleptic acceptance.

A trained panel of 24 panelists was formed, with an age range between 18 and 55 years, comprising students, homemakers, and professionals. To ensure evaluation reliability, participants were selected based on their documented prior experience in the sensory analysis of bee products, in accordance with the recommendations of the ISO 8586 standard for selected and expert sensory assessors [23]. Since the panel consisted of previously screened and trained evaluators, formal screening tests, such as basic taste recognition and olfactory threshold tests, were not repeated prior to this study. Furthermore, the panelists had previously received 120 h of training specifically tailored to perform propolis organoleptic analysis. The training covered the following topics: introduction to sensory analysis (definition, purpose, laboratory tour), fundamentals of sensory analysis (sight, smell, taste, touch, hearing), sensory perception, training in recognizing specific product stimuli, quantitative evaluation scales (descriptive and ranking), and the quantitative sensory evaluation process (blind tasting, structured evaluation forms, and inter-sample procedures). During training, the panel reached a consensus on descriptors after repeatedly evaluating propolis samples, with descriptor definitions adopted from the literature.

The panel size was determined following methodological criteria supported by scientific literature. The ISO 11132:2021 standard recommends forming quantitative descriptive panels of between eight and 12 evaluators to guarantee evaluation quality [24]. Nonetheless, various studies have documented the use of panels composed of 5 to 40 panelists to ensure reproducibility, sensitivity, and reliability of results [25]. Considering these precedents, a panel of 24 assessors was deployed for the present study.

The panel’s performance was validated using an analysis of variance (ANOVA) to assess consistency and homogeneity. The factors included in the model were the panelists, the samples, and their interaction. The results showed no significant differences among panelists (*p* = 0.1216), indicating that the assessors used the evaluation scale homogeneously. Furthermore, the absence of a significant panelist × sample interaction (*p* = 0.9217) confirms that the panel evaluated the samples consistently and reproducibly. Tukey’s multiple comparison test did not identify significant differences among panelists, corroborating the absence of outliers or inconsistent evaluators. Overall, this validation analysis demonstrated that the sensory panel was highly reproducible and generated a consensus in its evaluations. Specific details regarding panel validity are provided in Supplementary Table S2.

### 2.5. Evaluation Instrument and Scales

Each panelist individually evaluated the ten propolis samples over the course of three sessions, using two complementary instruments designed to capture both the descriptive characteristics and the overall acceptability:

Attribute-specific categorical scale: A descriptive categorical scale was applied, where perceived attributes were recorded without hedonic ranking. This methodology allowed for the determination of the presence or absence of specific characteristics in each sample without emitting affective judgments. The evaluated attributes were: (a) color: free description of the observed tone; (b) aroma: none, mild, medium, strong; (c) specific aroma notes: resinous, balsamic; (d) taste: insipid, sweet, bitter, salty, spicy; (e) consistency: soft, gummy, hard/rigid, malleable. This phase focused exclusively on descriptive sensory characterization attributes, excluding subjective liking assessments.

Nine-point hedonic Likert scale. Complementarily, a nine-point Likert-type hedonic scale was used to evaluate the degree of acceptance that each sample generated in the panelists. The attributes considered were overall acceptance, color, aroma, taste, and consistency. The nine-point hedonic scale included the following options:

1. Dislike extremely
2. Dislike very much
3. Dislike
4. Dislike slightly
5. Neither like nor dislike
6. Like slightly
7. Like
8. Like very much
9. Like extremely

These types of scales are widely validated in sensory studies due to their ability to capture differentiated levels of acceptance [26,27].

Given the current absence of a formal and standardized method specifically designed for the sensory analysis of raw propolis, the evaluation protocol was established by adapting approaches reported in previous studies on this product [28,29], complemented by generally accepted guidelines for sensory evaluation [30].

The samples were presented monadically, following a complete and balanced block design to avoid bias from sample presentation order. They were coded with random three-digit numbers and accompanied by their respective evaluation sheets. Participants were requested to abstain from ingesting food or drinks, smoking, or using oral products one hour before the test. During the session, to cleanse the palate between samples, evaluators rinsed their mouths with mineral water and consumed salt-free bread. Additionally, to prevent sensory fatigue and ensure accurate perception, the evaluation time per sample was 2 to 3 min, particularly for aroma, taste, and aftertaste. The rest interval between samples was 3 min due to the intensity of the product being tasted.

### 2.6. Statistical Analysis

The sensory data were analyzed through an integrated approach combining descriptive, non-parametric, and multivariate statistical techniques. Initially, a mean analysis was applied to the responses obtained from the categorical scale per attribute to identify frequencies and predominant patterns [31]. Regarding the results of the nine-point hedonic Likert scale, Spearman correlation coefficients were calculated to explore associations among the different sensory attributes [32]. This exploratory analysis highlights significant relationships between attributes but does not determine causality or specific drivers of acceptance.

Subsequently, a hierarchical cluster analysis (Ward’s method and squared Euclidean distance) was employed to classify the ten propolis samples into groups with similar sensory profiles [33]. For a detailed characterization of the resulting clusters, the means of the sensory attributes evaluated with the nine-point hedonic Likert scale were used.

To reduce data dimensionality and explore underlying relationships among the sensory attributes across samples, a Principal Component Analysis (PCA) was performed [34]. The suitability of the model was verified using the Kaiser-Meyer-Olkin (KMO) measure of sampling adequacy (with a threshold > 0.6) and Bartlett’s test of sphericity (requiring *p* ≤ 0.05).

Finally, because the sensory evaluations were recorded using a nine-point hedonic Likert scale (generating ordinal data), the non-parametric Kruskal–Wallis test was selected to compare the propolis samples across each evaluated attribute [35,36]. Statistically significant differences were considered at *p* ≤ 0.05. The sensory evaluation database was organized in Microsoft Excel (2025) version 16.94, and statistical analyses were executed using IBM SPSS Statistics software (version 23.0).

## 3. Results

### 3.1. Descriptive Sensory Profile of Propolis

For color evaluation, the Pantone color guide was used as a standardized visual reference. Although significant variations in individual perceptions emerged from the free descriptions of the observed tones—which prevented the establishment of a clear consensus among the panelists—three predominant chromatic categories were revealed: amber, dark, and reddish-brown. The dispersion in the responses indicates that a structured classification system is required to avoid ambiguity in the results; therefore, future studies should implement instrumental colorimetry.

**Figure 1:**
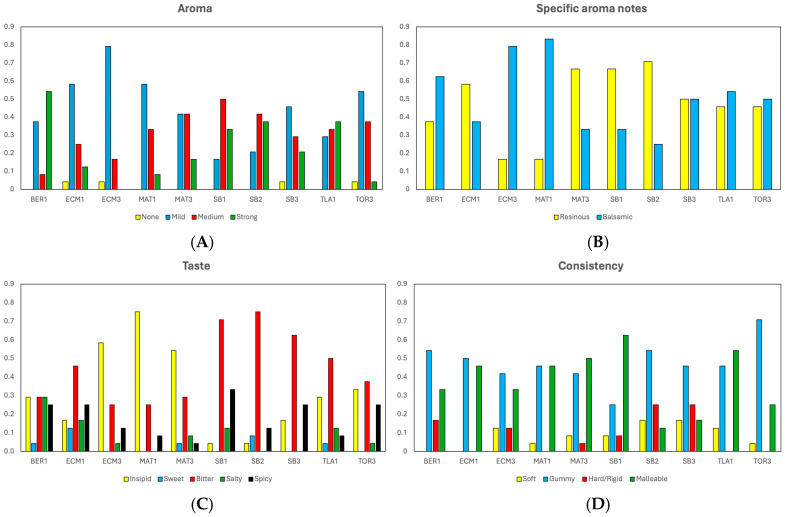
Means of attribute-specific categorical scale of the different propolis samples. (**A**) Aroma; (**B**) Specific aroma notes; (**C**) Taste; (**D**) Consistency. Abbreviations refer to locations and harvest number of the propolis samples. Standard deviations for all values are detailed in Supplementary Table S3.

Regarding the remaining attributes, Figure 1 illustrates that very few propolis samples were perceived as odorless. Samples BER1, SB2, and TLA1 presented medium to strong (intense) aromas, whereas ECM3, ECM1, and MAT1 stood out for their mild aromas. A clear distinction was observed between samples that evoked resinous notes associated with wood (e.g., SB2, SB1, and MAT3) and those with balsamic notes related to wax (e.g., MAT1 and ECM3).

The most predominant taste note was bitter, followed by insipid. A sweet taste was less prevalent, with only sample ECM1 standing out. Samples SB2 and SB1 recorded high scores for bitterness, whereas MAT1 and ECM3 were perceived as neutral or insipid. Regarding texture, a gummy consistency was most frequently observed in TOR3 and BER1. Conversely, samples SB1 and TLA1 were perceived as malleable, which is a desirable characteristic for the handling and practical application of propolis.

### 3.2. Sensory Correlations and Panel Preferences

Analysis using the Spearman correlation coefficient revealed significant associations among the evaluated sensory attributes (Table 1). To interpret these results, the strength of the correlation coefficients (r) was classified as weak (r < 0.3), moderate (0.3–0.5), or strong (r > 0.5). The analysis revealed a weak, albeit statistically significant, correlation (*r* = 0.162) between overall acceptance and taste. Given the low magnitude of this coefficient, its practical significance is minimal, indicating that taste alone does not drive the overall liking of the propolis extract. This is consistent with the inherent biological nature of the product; owing to its high concentration of polyphenols, flavonoids, and resins, the 10% (*w*/*v*) alcoholic extract presents challenging bitter, pungent, and astringent attributes. Consequently, overall acceptance is likely a multidimensional response rather than one dictated solely by taste.

Analysis using the Spearman correlation coefficient evidenced significant associations between the different sensory attributes (Table 1). To interpret the results, the strength of the correlation coefficients (r) was classified as weak (<0.03), moderate (0.3–0.5), or strong (>0.5).

Conversely, stronger correlations were observed among specific sensory properties. Notably, color showed a strong positive correlation with consistency (*r* = 0.589), as well as moderate correlations with taste (*r* = 0.460) and aroma (*r* = 0.378). In alcoholic propolis extracts, visual appearance is intrinsically linked to the concentration and type of dissolved botanical resins and waxes; a higher load of these compounds not only darkens the extract but also increases its viscosity and structural body (consistency). Thus, a denser visual perception often aligns with a more robust mouthfeel.

Finally, the strongest correlation in this study was found between taste and consistency (*r* = 0.629), followed closely by the correlation between taste and aroma (*r* = 0.578). This strong association can be attributed to the physical dynamics of the liquid matrix. The viscosity and body of the extract dictate how the concentrated sapid compounds interact with taste receptors during the 2-to-3-min evaluation period. A denser, more resinous liquid naturally coats the palate differently, leading to a more intense and lingering release of bitter and astringent notes. These robust correlations underscore that maximizing sensory acceptance requires an integrated formulation approach; optimizing taste alone is insufficient without achieving a balanced harmony among the extract’s physical body, visual cues, and olfactory attributes.

### 3.3. Sample Grouping Through Cluster Analysis and PCA

**Figure 2:**
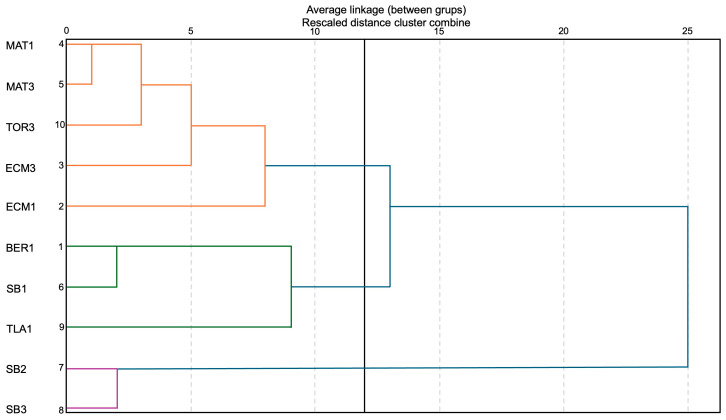
Hierarchical clustering of propolis samples. Dendrogram resulting from the cluster analysis based on the assessment of sensory attributes. The abbreviations indicate the location and the order of harvest of the propolis. The analysis was performed using the average linkage (Ward’s method and squared Euclidean distance).

To identify similarity patterns and classify the samples based on their sensory profiles, a hierarchical cluster analysis was performed. As shown in the dendrogram (Figure 2), this technique generated three clearly differentiated groups: cluster 1 comprised samples MAT1, MAT3, TOR3, ECM3, and ECM1; cluster 2 included BER1, SB1, and TLA1; and Cluster 3 consisted exclusively of samples SB2 and SB3.

To further characterize each group, Table 2 presents the mean scores of the sensory attributes organized by cluster. From these data, the following sensory profiles were defined:

Cluster 1. These samples recorded scores above the general mean for most attributes, except for aroma. They stood out particularly in color (5.64) and consistency (5.55), demonstrating that these samples maintain a sensorially acceptable appearance and texture. Since their overall acceptance (5.34) exceeded the general mean, this group exhibited the highest overall sensory acceptance.

Cluster 2. This group was distinguished by reaching the highest score for the aroma attribute (6.10), exceeding all other clusters and evaluated attributes. Likewise, this group recorded the maximum score for taste (5.26), positioning it as the group with the highest rating for this specific attribute. Although its overall acceptance remained above the general mean, the color attribute received a relatively low score (4.85). Overall, this profile is characterized by a strong aromatic perception and prominent taste, establishing it as the group with the most pronounced specific attributes.

Cluster 3. This group exhibited the lowest scores across all evaluated attributes, with notable decreases in overall acceptance (4.60), color (4.48), and taste (4.17), reflecting a lower sensory preference for the samples within this cluster.

Subsequently, PCA was performed. The suitability of the data for this analysis was confirmed by a Kaiser-Meyer-Olkin (KMO) sampling adequacy index of 0.643. Similarly, Bartlett’s test of sphericity was highly significant (*p* = 0.008), confirming the presence of correlations among the variables and justifying the application of the model. Consequently, two principal components with eigenvalues greater than 1.0 were extracted. The first component explained 58.7% of the total variance, while the second contributed an additional 27.4%, achieving a cumulative explained variance of 86.2% (Table 3).

**Figure 3:**
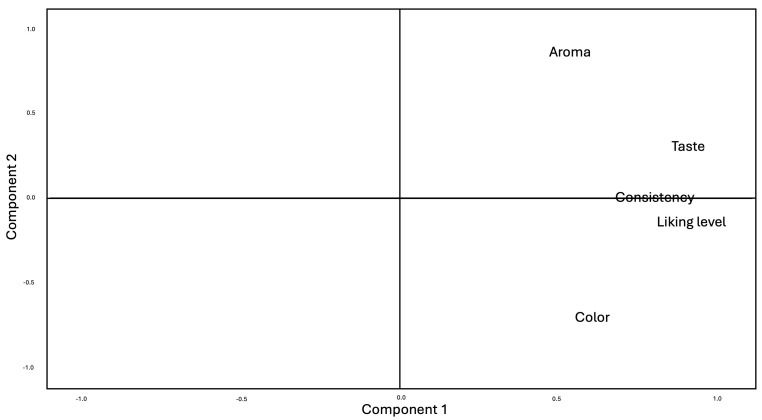
Arrangement of sensory attributes in the PCA factor plane. Graph showing the relationship between component 1 (overall acceptance, taste, consistency) and component 2 (aroma and color). Component 1 explains 58.704% of the variance and component 2 explains 27.449%, which represents 86.153% of the variance.

Analysis of the principal component loading matrix identified two structural groupings among the sensory attributes, graphically reflected in the factor space (Figure 3). Component 1 was primarily defined by consistency, overall acceptance, and color. Meanwhile, Component 2 showed a clear orientation toward aroma and taste, highlighting a marked opposition to the color attribute, as evidenced by the large geometric distance separating these vectors within the factor plane.

**Figure 4:**
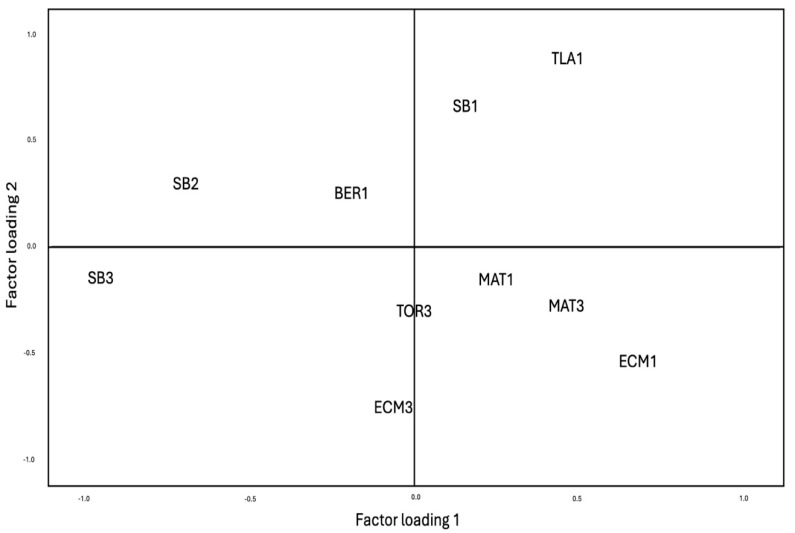
Classification of propolis samples in the PCA plane. Exhibits the spatial distribution of the samples according to component 1 and component 2, evidencing their grouping. Component 1 explains 58.704% of the variance and component 2 explains 27.449%, which represents 86.153% of the variance.

These patterns confirm that the panelists evaluated the sensory attributes distinctively across the different propolis samples. This differentiation is visualized in the PCA score plot (Figure 4), which illustrates the spatial distribution of the samples defined by the two principal components. Specifically, a clear grouping trend was observed for samples MAT1, MAT3, and ECM1 along the positive axis of Component 1. In contrast, samples SB1 and TLA1 were positioned predominantly along Component 2, which is characterized by high positive loadings for aroma and taste, and a negative loading for color.

These findings show close agreement with the cluster analysis results. Samples MAT1, MAT3, and ECM1 (Cluster 1) aligned with Component 1, reflecting their high scores for key attributes such as overall acceptance, color, and consistency. Conversely, the projection of samples SB1 and TLA1 onto Component 2 confirms their distinctive sensory profile, showing a strong association with aroma and taste, and recording the highest mean scores in the study (Table 2). However, as predicted by the component structure, these samples exhibited low mean scores for the color attribute, validating the negative loading exerted by color on this axis. The dispersion observed in Figure 4 for samples SB2 and SB3 (Cluster 3) showed a more isolated spatial distribution, evidencing a lower sensory preference or a distinct profile compared to the other propolis samples.

### 3.4. Statistical Differences Between Sensory Attributes and Propolis Samples

To determine whether the observed variability represented statistically significant differences among the samples, a Kruskal–Wallis test was performed (Table 4). The results confirmed that aroma (*p* = 0.010) and taste (*p* = 0.041) differed significantly among the samples (*p* ≤ 0.05). In contrast, overall acceptance, color, and consistency did not exhibit statistically significant variations.

Following the Kruskal–Wallis test, Dunn’s post hoc multiple comparison test was applied (detailed mean ranks and significant variations are presented in Supplementary Table S4). Regarding aroma, samples TLA1 and SB1 recorded the highest mean ranks, indicating superior sensory ratings for this attribute, whereas samples SB3 and ECM3 obtained the lowest ratings. Regarding taste, samples TLA1, ECM1, and SB1 were the highest rated. On the opposite extreme, SB3 and SB2 showed the lowest scores, reaffirming their lower sensory acceptance.

Overall, these results corroborate the previously observed trends. Samples TLA1 and SB1 consistently achieved high scores for aroma and taste, establishing them as the most preferred samples for these specific attributes, whereas SB3 and SB2 demonstrated lower sensory ratings. Crucially, since aroma and taste were significantly correlated with the remaining attributes (Table 1), optimizing these parameters could positively impact the global perception of the product. Consequently, prioritizing these parameters is recommended during the development or formulation stages of propolis-based products.

## 4. Discussion

The present study identified differentiating sensory patterns among various propolis samples. Initially, the descriptive characterization demonstrated that propolis from the Comarca Lagunera is distinguished by a predominantly bitter taste profile and an intense aroma. While the literature recognizes propolis as a valuable source of bioactive compounds, it has been documented that these intense sensory attributes can potentially compromise consumer acceptance [37].

Particularly, taste analysis showed a distinct trend toward bitter and insipid notes. These findings are consistent with those reported by Buitrago et al. [38], who recorded similar taste profiles when analyzing samples from various regions of Colombia. Likewise, the color evaluation agreed with previous research, such as that of Achenbach et al. [39], where this product is typically described as ranging from dark brown to reddish tones. Furthermore, Alcalá et al. [29] determined that propolis tends to exhibit a malleable consistency and that aroma represents one of the most critical attributes for consumers.

From this perspective, the aromatic intensity reported in the present study is supported by the intrinsic nature of propolis, which is characterized by the emission of strong and distinctive odors, categorized as either resinous (associated with wood) or balsamic (related to wax). This specific aromatic perception has been well-documented in official sources, such as the National Atlas of Bees and Bee Products [23].

The comprehensive analysis of the evaluated attributes and the differences observed among the samples support the hypothesis that the sensory profile of propolis may be influenced by its geographical origin, as well as by environmental factors such as climate and harvesting period [8,40,41]. However, since specific chemical or melissopalynological analyses were not conducted in this study, further research is required to objectively validate these relationships.

Consequently, there is an evident need to deepen sensory analysis through the implementation of standardized tools, such as Likert-type scales, which allow for the precise quantification of the perception of various attributes [26]. The application of these methodologies not only facilitates the detection of limiting characteristics but also strategically guides quality improvement processes and the formulation of new propolis-based products [28]. For example, one of the main challenges identified in the literature lies in the fact that incorporating propolis as a food additive generates intense aromas and bitter tastes that can compromise the palatability of the final product [42]. This phenomenon indicates that the primary obstacle to food supplementation with propolis is directly linked to its sensory attributes, particularly aroma and taste [28].

The results of this research reaffirm this premise, demonstrating that aroma and taste directly modulate sensory perception. Throughout the evaluations, these characteristics established themselves as determining factors, confirming that they are key properties governing the palatability and market acceptability of the product [12]. Although these attributes are critical, the importance of color, consistency, and overall acceptance should not be overlooked, as they also significantly affect global perception. In this regard, the results obtained using the nine-point hedonic Likert scale yielded scores ranged from four to six points, indicating intermediate acceptability.

These findings concur with those reported by Salamanca-Grosso et al. [43], who obtained scores within similar ranges during the sensory characterization of propolis. Furthermore, the recent literature exploring propolis from diverse geographical regions, such as Australia, highlights its tendency to exhibit specific and distinctive qualities [44]. Globally, propolis of varied origins—including African, Argentine, Brazilian, Canary Islands, Chilean, Indonesian, Japanese, and Nepalese—is often classified according to its primary botanical sources, geographical location, and climate [12]. While propolis is widely utilized in pharmaceutical and nutraceutical formulations where its quality and bioactivity are closely tied to these botanical origins and processing conditions [45], it is hypothesized that these unique environmental factors are also the primary drivers shaping the heterogeneous sensory profiles observed in this study.

In addition to these external environmental factors, the internal dynamics of sensory evaluation also play a crucial role. Specifically, the visual appearance of color was found to positively influence the scores allocated to aroma, taste, and consistency [46]. This interaction demonstrates that a favorable initial impression of color primes the evaluators, leading to a more positive assessment of the remaining sensory characteristics—a cognitive bias known as the halo effect [47]. In this context, color serves as a primary visual cue that establishes psychological expectations, which subsequently modulate and shape the actual gustatory experience. This interconnected perception is further supported by the significant relationships observed between aroma and the other attributes, highlighting that the evaluation of raw propolis relies on a complex process of multisensory integration rather than isolated sensory inputs [48].

To navigate this multisensory complexity, multivariate analyses such as hierarchical clustering and PCA methods have been widely used in previous research to distinguish and classify propolis samples based on their phytochemical content and biological properties [7,49]. Nonetheless, the application of these same techniques in this study allowed for effectively addressing the complexity of sensory data and establishing significant patterns among attributes, which is fundamental to achieving a comprehensive characterization of propolis.

Despite the value of these analytical tools, propolis has received limited attention in both academic research and commercial valuation compared to other bee products, such as honey and wax [12]. Although Mexico positions itself as one of the primary honey producers globally, it does not figure prominently among propolis-exporting countries; in fact, there is currently a lack of official public data regarding the trade of this resource in its raw form. This situation reflects an undervaluation of the product within both the national and international apicultural sectors.

In addition, most reported studies on propolis tend to focus primarily on its chemical composition and physicochemical properties [5,7,50,51]. These approaches are highly relevant, as it has been demonstrated that these properties are influenced by multiple factors, including botanical sources, bee genetics, hive structure, resource availability, environmental factors, and diseases [4].

However, regarding sensory evaluations, research is still in its infancy. When sensory studies are conducted, they commonly focus on food products in which propolis has been incorporated as a functional additive [13,15,52]. While such works are necessary to explore the functionality of propolis across different food matrices, there remains a notable scarcity of research dedicated exclusively to the sensory evaluation of propolis as a raw material.

Therefore, the study of raw propolis has not yet been fully explored, which opens important opportunities to investigate its sensory properties, both in its original form and in derived products.

Addressing this specific research gap, the present study focused strictly on the sensory profile of raw propolis. From the data obtained, it is hypothesized that the pronounced bitterness and intense aroma identified in the samples could be directly related to the local flora and the specific harvesting period [53,54]. Given that previous literature suggests geographical location can influence the specific compounds responsible for organoleptic characteristics [12,44], it can be inferred that sensory attributes might be shaped by these factors. Nevertheless, as this study did not include a compositional or botanical profile, these associations remain speculative and require further analytical investigation.

To translate these findings into practical applications and mitigate consumer rejection due to excessive bitterness and strong odors, sensory data can guide quality-improvement strategies at the apiary level [55]. Integrating standardized sensory protocols can facilitate a better understanding of the product’s organoleptic characteristics prior to market distribution.

Ultimately, the distinct sensory clusters identified in this study provide a robust framework for the practical classification and characterization of regional propolis strictly based on sensory perception. The establishment of specific sensory indices based on clustering patterns represents an initial step toward product standardization.

In practical terms, propolis from the Comarca Lagunera exhibits sensory attributes with clear areas susceptible to improvement, as evidenced by the evaluation with the Likert scale, but also demonstrates significant potential for commercial market integration. Future studies should consider analyzing the influence of consumer preferences and socioeconomic profiles [56,57], in addition to correlating sensory evaluations with comprehensive chemical and geographical profiles.

## 5. Conclusions

The present study identified a bitter taste and an intense aroma as the most prominent sensory attributes of propolis from the Mexican Comarca Lagunera, with statistically significant differences observed among the evaluated samples. Specifically, samples TLA1 and SB1 stood out due to their high scores in aroma and taste, demonstrating the highest sensory acceptability for these attributes, whereas samples SB2 and SB3 exhibited lower ratings across all evaluated parameters. Although these findings allow for the identification of favorable organoleptic profiles, this study presents exclusively sensory data, which are by themselves insufficient to substantiate a geographical indication or a conclusive regional differentiation, as chemical composition analyses were not conducted.

Therefore, the need to promote multidisciplinary research that complements this sensory evaluation with rigorous chemical characterization is emphasized. The integration of detailed molecular analyses is an indispensable requirement to understand the chemical basis underlying the properties of regional propolis, standardize its quality, and, ultimately, document its geographical differentiation and functional value with the scientific rigor necessary for strategic marketing.

## Figures and Tables

**Table 1:** Spearman correlations coefficients among evaluated sensory attributes.

Attributes		1 ^+^	2 ^+^	3 ^+^	4 ^+^	5 ^+^
1	Correlation	1	−0.024	−0.036	0.162 *	0.114
	Sig.	---	0.711	0.574	0.012	0.077
2	Correlation	−0.024	1	0.378 **	0.460 **	0.589 **
	Sig.	0.711	---	0.000	0.000	0.000
3	Correlation	−0.036	0.378 **	1	0.578 **	0.364 **
	Sig.	0.574	0.000	---	0.000	0.000
4	Correlation	0.162 *	0.460 **	0.578 **	1	0.629 **
	Sig.	0.012	0.000	0.000	---	0.000
5	Correlation	0.114	0.589 **	0.364 **	0.629 **	1
	Sig.	0.077	0.000	0.000	0.000	---

^+^ 1. Overall acceptance; 2. Color; 3. Aroma; 4. Taste; 5. Consistency. * Correlation is significant at the 0.05 level (two-tailed). ** Correlation is significant at the 0.01 level (two-tailed).

**Table 2:** Means of the sensory attributes grouped by cluster.

Clusters *	Overall Acceptance	Color	Aroma	Taste	Consistency
1	5.34	5.64	5.40	4.98	5.55
2	5.18	4.85	6.10	5.26	5.29
3	4.60	4.48	5.17	4.17	5.00
General mean	5.15	5.17	5.56	4.90	5.36

* Clusters classification: 1. MAT1, MAT3, TOR3, ECM3, and ECM1; 2. BER1, SB1, and TLA1; 3. SB2 and SB3.

**Table 3:** Total variance explained and PCA model adequacy.

Component	Eigenvalue	% of Variance Explained	% Cumulative
1 (Factor 1)	2.935	58.704	58.704
2 (Factor 2)	1.372	27.449	86.153
3	0.449	8.972	95.125
4	0.146	2.923	98.048
5	0.098	1.952	100.000
KMO			0.643
Bartlett’s test of sphericity			23.779
df (degrees of freedom)			10
*p*-value			<0.008

**Table 4:** Kruskal–Wallis test results for sensory attributes across propolis samples.

Attribute	Chi-Square	Degrees of Freedom	*p*-Value *
Overall acceptance	8.541	9	0.481
Color	16.457	9	0.058
Aroma	21.702	9	0.010 *
Taste	17.498	9	0.041 *
Consistency	8.560	9	0.479

* Statistical significance *p* ≤ 0.05.

## Data Availability

The original contributions presented in this study are included in the article/Supplementary Materials. Further inquiries can be directed to the corresponding authors.

## Supplementary Materials

The following supporting information can be downloaded at: https://www.mdpi.com/article/10.3390/foods15142450/s1, Table S1: Specific conditions of each collected sample; Table S2: Validity of the sensory panel; Table S3: Means and standard deviations of sensory attributes by propolis sample; Table S4: Dunn’s post hoc test according to evaluated attributes.
