# Supplementary material for: Sensory Variability in Propolis from the Mexican Comarca Lagunera: A Multivariate Analysis
Source: Foods. 2026 Jul 10;15(14):2450. doi: 10.3390/foods15142450 (PMC13408831; doi:10.3390/foods15142450)
Supplement: Supplementary file 1 [file foods-15-02450-s001.zip › foods-4401216-supplementary.pdf]

Supplementary Table S1.

Specific conditions of each collected sample

ID	Geographical origin	Harvest*	Collection dates	Min. Temp. (°C)	Max. Temp. (°C)	Precipitation (mm)
BER1	Bermejillo, Mapimí	1	February 2024 to April 2024*	9.9	31.1	0
ECM1	El Coyote, Matamoros	1	February 2024 to April 2024	14.7	30.1	0
ECM3	El Coyote, Matamoros	3	November 2024 to February 2025	10.4	26.8	0
MAT1	Matamoros	1	February 2024 to April 2024	14.1	30.3	0
MAT3	Matamoros	3	November 2024 to February 2025	10.2	25.0	0
SB1	Simón Bolívar	1	February 2024 to April 2024	13.1	25.5	0
SB2	Simón Bolívar	2	May 2024 to September 2024	10.5	33.6	1.5
SB3	Simón Bolívar	3	November 2024 to February 2025	7.9	31.3	0
TLA1	Tlahualilo	1	February 2024 to April 2024	9.9	31.2	0
TOR3	Torreón	3	November 2024 to February 2025	10.4	26.8	0

Abbreviations indicate the location and the order of harvest. *Propolis samples were grouped into three harvest periods: 1 (Mesquite/spring), 2 (Multifloral/summer), and 3 (Multifloral/autumn-winter).

Supplementary Table S2.

Validity of the sensory panel

ANOVA reproducibility - Panelists vs. Samples				
Source	DF	Mean Square	F-Valor	Pr > F
Panelist	23	4.4544928	4.45	0.1216
Sample	2	0.6843972	0.68	0.5690
Panelist*Sample	46	0.4136232	0.41	0.9217
Eigenvalues of the correlation matrix				
	Eigenvalue	Difference	Proportion	Cumulative
1	5.58084863	3.40912528	0.6201	0.6201
2	2.17172335	1.16548825	0.2413	0.8614
Tukey's Studentized Range Test (HSD)				
Alpha		0.05		
Error Degrees of Freedom		3		
Mean Square Error		1		
Critical Value of Studentized Range		11.68113		

Supplementary Table S3.

Means and standard deviations of sensory attributes by propolis sample

Attributes	BER1	ECM1	ECM3	MAT1	MAT3	SB1	SB2	SB3	TLA1	TOR3
None	0.000 ± 0.000	0.042 ± 0.204	0.042 ± 0.204	0.000 ± 0.000	0.000 ± 0.000	0.000 ± 0.000	0.000 ± 0.000	0.042 ± 0.204	0.000 ± 0.000	0.042 ± 0.204
Mild	0.375 ± 0.495	0.583 ± 0.504	0.792 ± 0.415	0.583 ± 0.504	0.417 ± 0.504	0.167 ± 0.381	0.208 ± 0.415	0.458 ± 0.509	0.292 ± 0.464	0.542 ± 0.509
Medium	0.083 ± 0.282	0.250 ± 0.442	0.167 ± 0.381	0.333 ± 0.482	0.417 ± 0.504	0.500 ± 0.511	0.417 ± 0.504	0.292 ± 0.464	0.333 ± 0.482	0.375 ± 0.495
Strong	0.542 ± 0.509	0.125 ± 0.338	0.000 ± 0.000	0.083 ± 0.282	0.167 ± 0.381	0.333 ± 0.482	0.375 ± 0.495	0.208 ± 0.415	0.375 ± 0.495	0.042 ± 0.204
Resinous	0.375 ± 0.495	0.583 ± 0.504	0.167 ± 0.381	0.167 ± 0.381	0.667 ± 0.482	0.667 ± 0.482	0.708 ± 0.464	0.500 ± 0.511	0.458 ± 0.509	0.458 ± 0.509
Balsamic	0.625 ± 0.495	0.375 ± 0.495	0.792 ± 0.415	0.833 ± 0.381	0.333 ± 0.482	0.333 ± 0.482	0.250 ± 0.442	0.500 ± 0.511	0.542 ± 0.509	0.500 ± 0.511
Insipid	0.292 ± 0.464	0.167 ± 0.381	0.583 ± 0.504	0.750 ± 0.442	0.542 ± 0.509	0.042 ± 0.204	0.042 ± 0.204	0.167 ± 0.381	0.292 ± 0.464	0.333 ± 0.482
Sweet	0.042 ± 0.204	0.125 ± 0.338	0.000 ± 0.000	0.000 ± 0.000	0.042 ± 0.204	0.000 ± 0.000	0.083 ± 0.282	0.000 ± 0.000	0.042 ± 0.204	0.000 ± 0.000

Bitter	0.292 ± 0.464	0.458 ± 0.509	0.250 ± 0.442	0.250 ± 0.442	0.292 ± 0.464	0.708 ± 0.464	0.750 ± 0.442	0.625 ± 0.495	0.500 ± 0.511	0.375 ± 0.495
Salty	0.292 ± 0.464	0.167 ± 0.381	0.042 ± 0.204	0.000 ± 0.000	0.083 ± 0.282	0.125 ± 0.338	0.000 ± 0.000	0.000 ± 0.000	0.125 ± 0.338	0.042 ± 0.204
Spicy	0.250 ± 0.442	0.250 ± 0.442	0.125 ± 0.338	0.083 ± 0.282	0.042 ± 0.204	0.333 ± 0.482	0.125 ± 0.338	0.250 ± 0.442	0.083 ± 0.282	0.250 ± 0.442
Soft	0.000 ± 0.000	0.000 ± 0.000	0.125 ± 0.338	0.042 ± 0.204	0.083 ± 0.282	0.083 ± 0.282	0.167 ± 0.381	0.167 ± 0.381	0.125 ± 0.338	0.042 ± 0.204
Gummy	0.542 ± 0.509	0.500 ± 0.511	0.417 ± 0.504	0.458 ± 0.509	0.417 ± 0.504	0.250 ± 0.442	0.542 ± 0.509	0.458 ± 0.509	0.458 ± 0.509	0.708 ± 0.464
Hard/Rigid	0.167 ± 0.381	0.000 ± 0.000	0.125 ± 0.338	0.000 ± 0.000	0.042 ± 0.204	0.083 ± 0.282	0.250 ± 0.442	0.250 ± 0.442	0.000 ± 0.000	0.000 ± 0.000
Malleable	0.333 ± 0.482	0.458 ± 0.509	0.333 ± 0.482	0.458 ± 0.509	0.500 ± 0.511	0.625 ± 0.495	0.125 ± 0.338	0.167 ± 0.381	0.542 ± 0.509	0.250 ± 0.442

Abbreviations indicate the location and the order of harvest.

Supplementary Table S4.

Dunn's post hoc test according to evaluated attributes

Samples	Aroma	Flavor
BER1	135.020	128.000
ECM1	101.750	139.620
ECM3	93.500	111.940
MAT1	120.900	120.150
MAT3	122.560	132.250
SB1	147.440	133.040
SB2	116.750	91.420
SB3	92.100	90.000
TLA1	156.980	147.810
TOR3	118.000	110.670

Abbreviations indicate the location and the order of harvest.
